# Sweet Secrets: Exploring Novel Glycans and Glycoconjugates in the Extracellular Polymeric Substances of “*Candidatus* Accumulibacter”

**DOI:** 10.1021/acsestwater.4c00247

**Published:** 2024-07-12

**Authors:** Timothy Páez-Watson, Sergio Tomás-Martínez, Roeland de Wit, Sunanda Keisham, Hiroaki Tateno, Mark C. M. van Loosdrecht, Yuemei Lin

**Affiliations:** † Department of Biotechnology, Delft University of Technology, Van der Maasweg 9, Delft 2629 HZ, The Netherlands; ‡ Cellular and Molecular Biotechnology Research Institute, National Institute of Advanced Industrial Science and Technology (AIST), Central 6, 1-1-1 Higashi, Tsukuba, Ibaraki 305-8566, Japan

**Keywords:** glycans, glycoproteins, glycomics, extracellular polymeric substances, lectin microarray

## Abstract

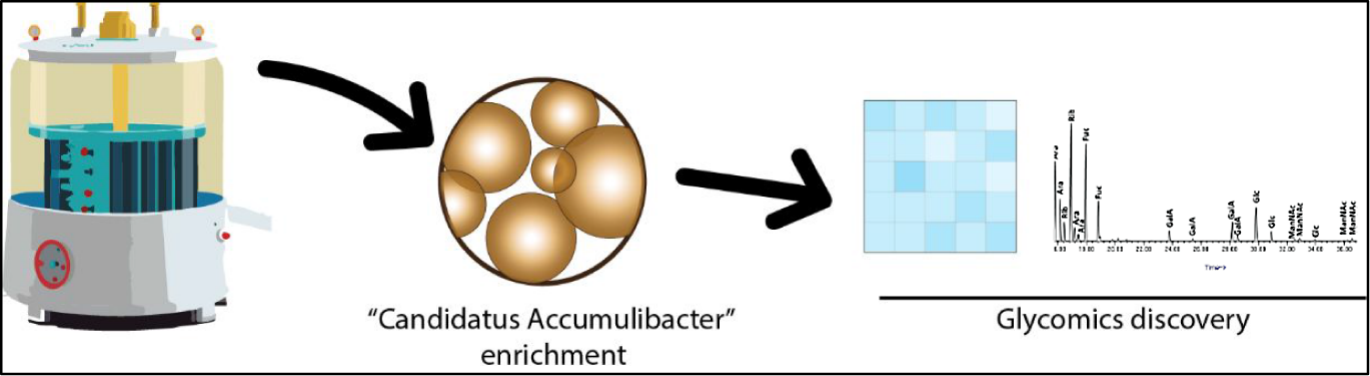

Biological wastewater treatment relies on microorganisms that grow as flocs, biofilms, or granules for efficient separation of biomass from cleaned water. This biofilm structure emerges from the interactions between microbes that produce, and are embedded in, extracellular polymeric substances (EPS). The true composition and structure of the EPS responsible for dense biofilm formation are still obscure. We conducted a *bottom-up* approach utilizing advanced glycomic techniques to explore the glycan diversity in the EPS from a highly enriched “*Candidatus* Accumulibacter” granular sludge. Rare novel sugar monomers such as N-Acetylquinovosamine (QuiNAc) and 2-O-Methylrhamnose (2-OMe-Rha) were identified to be present in the EPS of both enrichments. Further, a high diversity in the glycoprotein structures of said EPS was identified by means of lectin based microarrays. We explored the genetic potential of “*Ca*. Accumulibacter” high quality metagenome assembled genomes (MAGs) to showcase the shortcoming of *top-down* bioinformatics based approaches at predicting EPS composition and structure, especially when dealing with glycans and glycoconjugates. This work suggests that more *bottom-up* research is necessary to understand the composition and complex structure of EPS in biofilms since genome based inference cannot directly predict glycan structures and glycoconjugate diversity.

## Abstract 2

Unveiling novel glycans in ″*Ca*. Accumulibacter” EPS elucidates crucial insights into environmental bacterial interactions, influencing wastewater treatment efficiency and microbial community dynamics.

## Introduction

Biological wastewater treatment relies on microbial communities that form aggregates called biofilms, flocs or granules, which play a pivotal role in the separation of biomass from treated water.^1,2^ These structures house microorganisms embedded within a complex mixture of extracellular polymeric substances (EPS), which are produced by the microorganisms themselves.^3^ Despite the intricate nature of EPS, significant progress has been made by focusing research on community members that are easily controllable in lab reactors. Among these organisms, “*Candidatus* Accumulibacter”, a well-studied Gram-negative bacterium, emerges as a dominant member in most aerobic granular sludge (AGS) systems^4^ and is believed to play a major role in EPS formation. Despite not being isolated as a pure culture, “*Ca*. Accumulibacter” can be highly enriched in open lab cultures while maintaining the desired biofilm granular structure. Consequently, “*Ca.* Accumulibacter” has become a valuable model organism to study not only EPS formation but also the functioning, relationships, and assembly of microbial aggregates more broadly.

EPS plays a pivotal role in biofilm formation,^5^ provides protection against predation and environmental stress,^6^ facilitates nutrient cycling,^7^ and shapes overall microbial community structure.^8^ Their composition, exceedingly complex, emerges from active secretion, cell decay and sorption from the environment.^9^ Thus, they comprise of sugars, proteins, nucleic acids and lipids, although the reported composition is strongly dependent on the method employed for its extraction^10^ and analysis.^11^ Focusing on isolating EPS into their individual molecular components overlooks the potential existence of combinations of these molecules. In this context, “glycans”, which denote sugar chains, can be found as free molecules or linked to other macromolecules, particularly proteins and lipids.

Glycans are some of the most complex macromolecules in nature. Not only are their basic components diverse (typically ranging from 3 to 7 carbons) but the types of linkages (i.e., glycosidic bonds) that can occur at each individual carbon leads to different degrees of branching resulting in a nearly unlimited range of structures.^8,12^ In addition, in a microbial community each individual member could contribute to a unique set of glycan molecules which further hinders the understanding of the EPS’s glycome. Thus, developing systematic methods to better understand the sugar component that determines the EPS of a biofilm is of paramount importance. One such method is a “top down*”* approach in which the genetic makeup of microbial communities can be analyzed and the potential for production of glycans predicted.^13^ This method, however, is limited to a set of well-studied polysaccharides and lacks the discovery of novel or unknown structures. We propose a different method which involves a “bottom up” approach to start with examining the glycan composition (i.e., *what is there)* to guide the further analysis on a species-based proteomic or genomic analysis.

Recent advances in next-generation mass spectrometry and an ever growing resolution have revolutionized our ability to explore the composition of glycans from environmental samples.^14−16^ The high precision and sensitivity allow for the identification of novel glycans. By employing these cutting-edge techniques, researchers are now equipped to identify and characterize new glycan structures within the EPS of “*Ca.* Accumulibacter”, this way expanding our knowledge of the glycan diversity in these bacteria. High throughput techniques such as lectin microarrays^17^ exploit the natural selectivity of lectins to recognize specific glycan structures. Recently, the use of this technique was combined with protein identification, opening the possibility to study glycoconjugates such as glycoproteins.

Glycoproteins in bacteria have only recently gained scientific attention, as glycosylation was long believed to be exclusive to eukaryotic organisms.^18,19^ However, pathogenic bacteria have been found to contain multiple glycoproteins that play significant roles in various processes, for example the bacterial adhesion to host mucosal membranes.^20^ In addition, an array of glycoproteins were recently discovered in bacteria from environmental samples, e.g., from an enrichment of anaerobic ammonium oxidizing (ANAMMOX) bacteria^14,21^ indicating not only their presence but also their high variety. Consequently, it is crucial to continue investigating the presence of glycoproteins in environmental bacteria and explore their potential connections to the formation and function of EPS.

Glycoproteins result from protein glycosylation, a post-translational modification that influences protein structure, stability, and functionality. Two primary protein glycosylation systems have been identified in bacteria: *en-bloc* and *sequential* glycosylation.^19^*En-bloc* glycosylation involves the assembly of a lipid-oligosaccharide in the cytoplasmic membrane, followed by the export and transfer to a protein in the extracellular space.^22,23^ Conversely, sequential glycosylation entails the stepwise transfer of sugar moieties (mono or oligosaccharides) onto proteins.^24^ While extensive information exists regarding these processes in model organisms like *Campylobacter jejuni* and *Haemophilus influenzae*, limited knowledge is available concerning protein glycosylation mechanisms in microorganisms commonly found in wastewater treatment plants, such as ″*Ca.* Accumulibacter”.

In this paper, we aim to uncover the functional significance of glycoproteins and their associated glycans within the EPS of *“Ca.* Accumulibacter”. For this, we adopted a comprehensive bottom-up approach to investigate the diversity of the glycome within the EPS of ″*Ca.* Accumulibacter.″ Utilizing advanced glycomic techniques, we identified previously elusive novel glycan structures and explored the variety of glycoproteins present in two highly enriched “*Ca.* Accumulibacter” granular cultures. Guided by these results, we examined the genetic potential of available genomes of “*Ca.* Accumulibacter” for the production of novel glycans and glycoproteins. This work highlights the importance of a thorough analysis of structural components of EPS rather than relying solely on functional roles from genomic-only inferred components.

## Materials and Methods

### Reactor Operation

Two reactor conditions were tested for this research. Both reactors were operated under the exact same conditions except for a change in the stirring speed of the reactor impeller (400 vs 800 rpm) to enrich different sized granules. The “*Ca.* Accumulibacter” enrichment was obtained in a 2 L (1.5 L working volume) sequencing batch reactor (SBR), following conditions similar to the one described by Guedes da Silva et al.^200^ with some adaptations. The reactor was inoculated by using activated sludge from a municipal wastewater treatment plant (Harnaschpolder, The Netherlands). Each SBR cycle lasted 6 h, consisting of 30 min of settling, 50 min of effluent removal, 10 min of N_2_ sparging, 5 min of feeding, 130 min of anaerobic phase and 135 min of aerobic phase. The hydraulic retention time (HRT) was 12 h (removal of 750 mL of broth per cycle). The average solid retention time (SRT) was controlled to 8 days by the removal of effluent at the end of the mixed aerobic phase. The pH was controlled at 7.0 ± 0.1 by dosing 1 M HCl or 1 M NaOH. The temperature was maintained at 20 ± 1 °C.

The reactor was fed with two separate media: a concentrated COD medium (400 mg COD/L) of acetate (17 g/L NaAc × 3H_2_O) and a concentrated mineral medium (1.53 g/L NH_4_Cl, 1.59 g/L MgSO_4_ × 7H_2_O, 0.40 g/L CaCl_2_ × 2H_2_O, 0.48 KCl, 0.04 g/L N-allylthiourea (ATU), 2.22 g/L NaH_2_PO_4_ × H_2_O, 0.04 g/L yeast extract and 6 mL/L of trace element solution prepared following Smolders et al.^201^ In each cycle, 75 mL of each medium was added to the reactor together with 600 mL of demineralized water. The final feed contained 400 mg of COD/L of acetate. Extracellular concentrations of phosphate and ammonium were measured with a Gallery Discrete Analyzer (Thermo Fisher Scientific, Waltham, MA). Acetate was measured by high performance liquid chromatography (HPLC) with an Aminex HPX-87H column (Bio-Rad, Hercules, CA), coupled to RI and UV detectors (Waters, Milford, MA), using 0.0015 M phosphoric acid as eluent supplied at a flow rate of 1 mL/min.

### Microbial Community Analysis

The microbial community of each reactor condition was characterized after a minimum of 4 residence times was reached (approximately 35 days of operation). Two orthogonal approaches were used for community characterization: 16S amplicon sequencing and Fluoresence In Situ Hybdirization (FISH).

For 16S RNA amplicon sequencing, DNA was extracted from the granules using the DNeasy UltraClean Microbial kit (Qiagen, Venlo, The Netherlands), using the manufacturer’s protocol. The extracted DNA was quantified using a Qubit 4 instrument (Thermo Fisher Scientific, Waltham, MA). Samples were sent to Novogene Ltd. (Hong Kong, China) for amplicon sequencing of the V3-4 hypervariable region of the 16S rRNA gene (position 341-806) on a MiSeq desktop sequencing platform (Illumina, San Diego, CA) operated in paired-end mode. The raw sequencing reads were processed by Novogene Ltd. (Hong Kong, China) and quality filtered using the QIIME software.^25^ Chimeric sequences were removed using UCHIME^26^ and sequences with ≥97% identity were assigned to the same operational taxonomic units (OTUs) using UPARSE.^27^ Each OTU was taxonomically annotated using the Mothur software against the SSU rRNA database of the SILVA Database.^28^ Sequences obtained are deposited under Bioproject accession number PRJNA1084229 in the NCBI database.

For FISH, samples underwent the procedures outlined by^29^ for handling, fixation, and staining. Bacteria were selectively identified using a blend of EUB338, EUB338-II, and EUB338-III probes.^30,31^ ″*Ca.* Accumulibacter” was visualized employing a mixture of PAO462, PAO651, and PAO846 probes (referred to as PAOmix).^32^ Hybridized samples were subsequently examined utilizing an Axio Imager 2 fluorescence microscope (Zeiss, Oberkochen, Germany). To quantify and analyze the fluorescent pixels in the microscopic images, a custom image analysis tool was developed. The tool employs algorithms to identify and quantify different color categories, including blue (Eubacteria only), purple (PAOmix + Eubacteria), and green (GAOmix + Eubacteria) providing a comprehensive analysis of the microbial composition. The tool is available on GitHub [https://github.com/TP-Watson/FISH-quantification-PaezWatson].

### EPS Extraction and Characterization

#### EPS Extraction from the Biomass

Biomass samples collected at the end of the aerobic phase were freeze-dried prior to EPS extraction. EPS were extracted in alkaline conditions at high temperature, using a method adapted from Felz et al.^10^ Freeze-dried biomass were stirred in of 0.1 M NaOH (1% w/v of volatile solids) at 80 °C for 30 min. Extraction mixtures were centrifuged at 4000xg at 4 °C for 20 min. Supernatants were collected and dialyzed overnight in dialysis tubing with a molecular cutoff of 3.5 kDa, frozen at −80 °C and freeze-dried. The freeze-dried extracted EPS samples were stored for further analysis.

#### Determination of the Total Protein and Carbohydrate Contents of the Extracted EPS

The total protein content was estimated using the bicinchoninic acid (BCA) assay^33^ with bovine serum albumin (BSA) as standard. The total carbohydrate content was determined using the phenol–sulfuric acid assay^34^ with glucose as standard. Both analyses were performed as described by.^11^

### Glycosyl Composition and Detection of Glycoproteins in the EPS

#### Glycosyl Composition Analysis by TMS Method

Glycosyl composition analysis of the extracted EPS was performed at the Complex Carbohydrate Research Center (CCRC, the University of Georgia) by combined GC/MS of the O-trimethylsilyl (TMS) derivatives of the monosaccharide methyl glycosides produced from the sample by acidic methanolysis. These procedures were carried out as previously described in ref^15^. In brief, lyophilized EPS aliquots of 300 μg were added to separate tubes with 20 μg of inositol as the internal standard. Methyl glycosides were then prepared from the dry sample following the mild acid treatment by methanolysis in 1 M HCl in methanol at 80 °C (16 h). The samples were re-N-acetylated with 10 drops of methanol, 5 drops of pyridine, and 5 drops of acetic anhydride and were kept at room temperature for 30 min (for detection of amino sugars). The sample was then per-o-trimethylsilylated by treatment with Tri-Sil (Pierce) at 80 °C (30 min). These procedures were carried out as described by.^35^ GC/MS analysis of the per-o-trimethylsilyl methyl glycosides was performed on an AT 7890A gas chromatograph interfaced to a 5975B MSD mass spectrometer using a Supelco EC-1 fused silica capillary column (30 m × 0.25 mm ID) and the temperature gradient shown in Table 1.

**Table 1:** Temperature Program for GC-MS Analysis for the TMS Method

	rate (°C/min)	value (°C)	hold time (min)	run time (min)
initial		80	2	2
Ramp 1	20	140	2	7
Ramp 2	2	200	0	37
Ramp 3	30	250	5	43.7

#### Identification of Methylated Sugar by Alditol Acetates

Identification of methylated sugar was performed by GC-MS of the alditol acetates as described.^16^ The analysis was performed on 400 mg of the sample. The sample was hydrolyzed in 2 M trifluoroacetic acid (TFA) for 2 h in a sealed tube at 120 °C, reduced with NaBD_4_, and acetylated using acetic anhydride/TFA. The resulting alditol acetates were analyzed on an Agilent 7890A GC (Table 2) interfaced to a 5975C MSD, electron impact ionization mode. A SP2331 fused silica capillary was used as a column.

**Table 2:** Temperature Program for GC-MS Analysis by Alditol Acetates

	rate (°C/min)	value (°C)	hold time (min)	run time (min)
initial		60	1	1
Ramp 1	27.5	170	0	5
Ramp 2	4	235	2	23.5
Ramp 3	3	240	12	36.9

#### Glycoproteins Detection by Lectin Microarray

The high-density lectin microarray was constructed based on the procedure outlined by.^17^ To label EPS, 0.4 μg of it was mixed with Cy3-*N*-hydroxysuccinimide ester (GE Healthcare). Excess Cy3 was removed by using Sephadex G-25 desalting columns (GE Healthcare). The Cy3-labeled EPS was then diluted to a concentration of 0.5 μg/mL with probing buffer, which contained 25 mM Tris-HCl (pH 7.5), 140 mM NaCl, 2.7 mM KCl, 1 mM CaCl_2_, 1 mM MnCl_2_, and 1% Triton X-100. The mixture was incubated with the lectin microarray overnight at 20 °C. The lectin microarray was washed three times with probing buffer, and the resulting fluorescence images were acquired using a Bio-Rex scan 200 evanescent-field-activated fluorescence scanner (Rexxam Co. Ltd., Kagawa, Japan).

### Gene Identity Analysis

Genomic analysis was undertaken to explore the existence of genes within various ″*Ca.* Accumulibacter” species that are associated with potential glycan synthesis and protein glycosylation machinery. We acquired MAG (Metagenome-Assembled Genome) sequences for 19 ″*Ca.* Accumulibacter” species from the European Nucleotide Archive as described in ref^36^. BLAST analysis was executed on the coding sequences of these genomes to identify the presence of (or potential for) specific genes in a reference set (reference genes used in Table S2). Sequence alignment was employed to evaluate conservation and recognize potential orthologues or homologues (min_identity 30%, evalue e-12).

## Results

### Reactors Performance and Microbial Community

**Figure 1:**
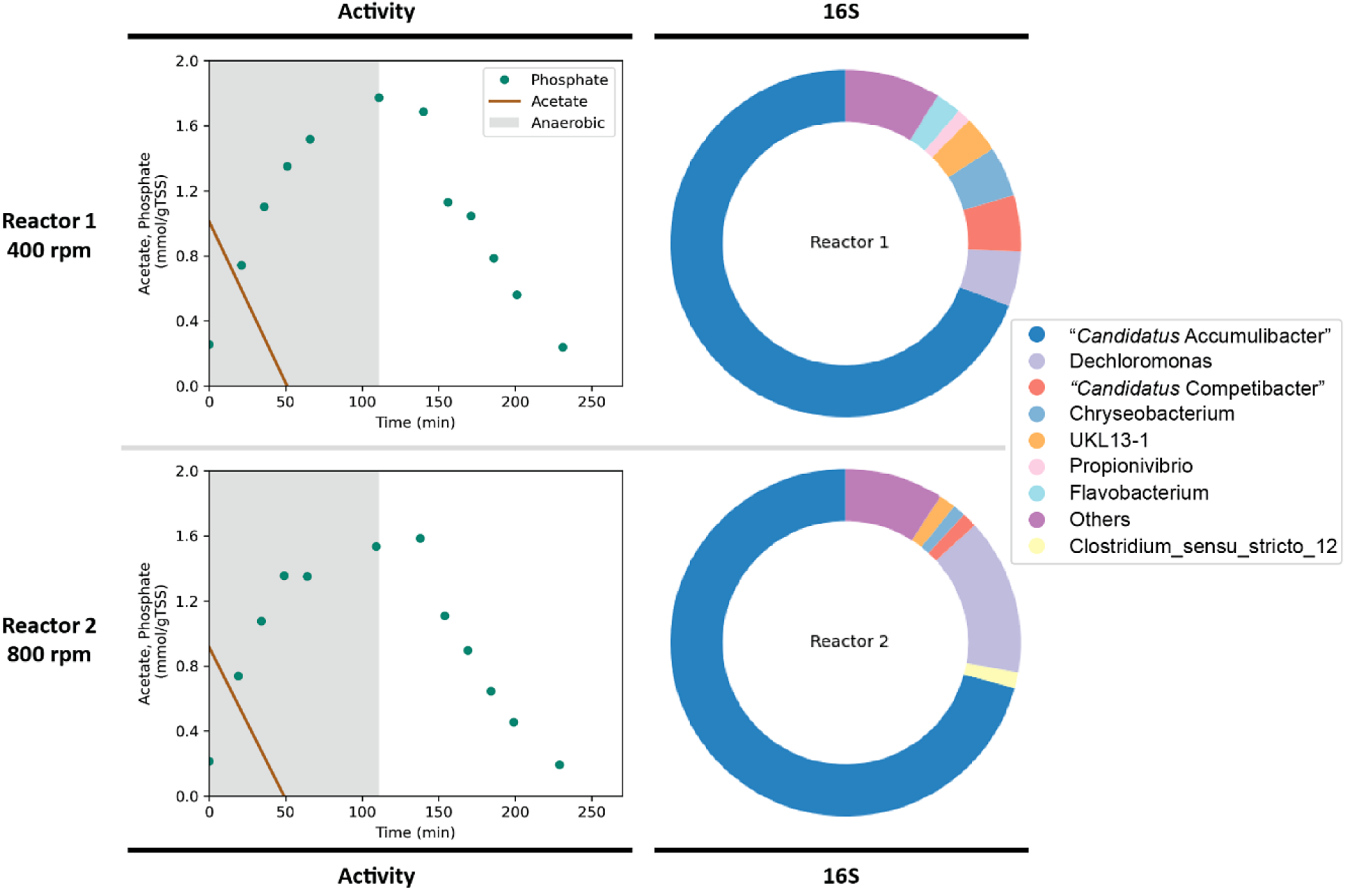
Reactor characteristics for the enrichments with the impeller rotating at 400 (top) and 800 (bottom) rpm at steady state. Each panel presents the activity test of a cycle by showing the concentrations of phosphate and acetate (mmol/gTSS) (left) and the microbial community abundance based on 16S rRNA amplicon sequencing (right). Both activity tests indicate that acetate was taken up during the anaerobic phase with the concurrent release of phosphate, typical for PAOs enrichments. For 16S rRNA results, the resolution at genus level indicates ≥1% abundance, otherwise genus with <1% abundance were clustered into the category “Others”.

Two reactor enrichments were operated under the same conditions except for the rotational speed of the impeller (reactor 1:400 rpm; reactor 2:800 rpm). Both enrichments achieved a steady state in which the cyclic profiles of phosphate and acetate concentrations were typical of a polyphosphate accumulating organism (PAOs) enrichment (Figure 1: Activity).^37^ Biomass concentrations in both reactors were relatively comparable at 4.61 ± 0.05 and 4.68 ± 0.08 g/L total suspended solids (TSS) for reactors 1 and 2, respectively. Further reactor characterization revealed a closely related microbial community based on 16S rRNA gene analysis (Figure 1: 16S), in both cases dominated by the genus *“Ca.* Accumulibacter”. These findings align with the FISH results, indicating a strong dominance of “*Ca.* Accumulibacter” in both enrichments (95.8 ± 4.4% and 97.9 ± 2.3% of biovolume in reactor 1 and 2, respectively—Supplementary Figure).

### EPS Yield and Characterization

**Figure 2:**
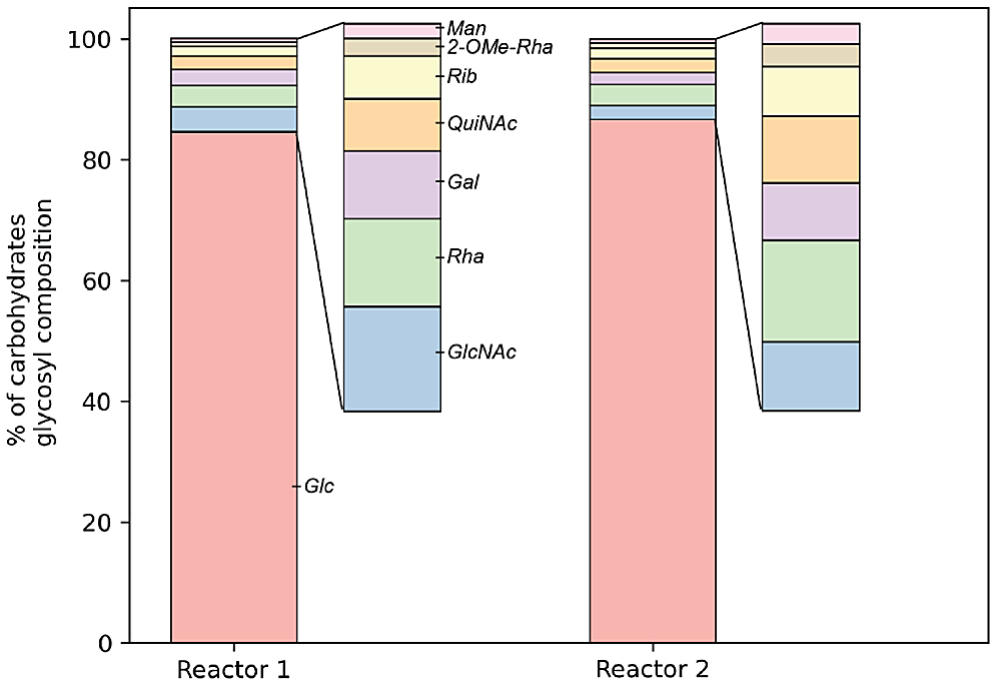
Glycosyl composition of the extracted EPS as relative mole abundance from the total amount of carbohydrate monomers determined by GC-MS. Carbohydrate monomers detected: glucose (Glc), Rhamnose (Rha), Mannose (Man), Galactose (Gal), Ribose (Rib,) N-Acetylglucosamine (GlcNAc), N-Acetylquinovosamine (QuiNAc), and 2-O-Methylrhamnose (2-OMe-Rha).

To characterize the “glycans” in the EPS of the “*Ca.* Accumulibacter” enrichment, the biomass from each reactor was collected at the end of the aerobic phase, and the EPS were extracted. The total carbohydrate content was determined as 50.6 mg_eq_glucose_/g_EPS_ for reactor 1 and 64.1 mg_eq_glucose_/g_EPS_ for reactor 2. Additionally, the total protein content was determined as 288.9 mg_eq_BSA_/g_EPS_ for reactor 1 and 398.6 mg_eq_BSA/_g_EPS_ for reactor 2. Analysis of the specific glycosyl composition of the EPS (Figure 2, GC-MS spectrum in Supporting Information) revealed a similar glycan profile, with the presence of both common carbohydrate monomers such as glucose (Glc), rhamnose (Rha), 2-O-Methylrhamnose (2-OMe-Rha), mannose (Man), galactose (Gal), ribose (Rub), N-Acetylglucosamine (GlcNAc), and relatively uncommon monomer N-Acetylquinovosamine (QuiNAc).

### Potential for Biosynthesis of the Carbohydrate Monomer QuiNac

**Figure 3:**
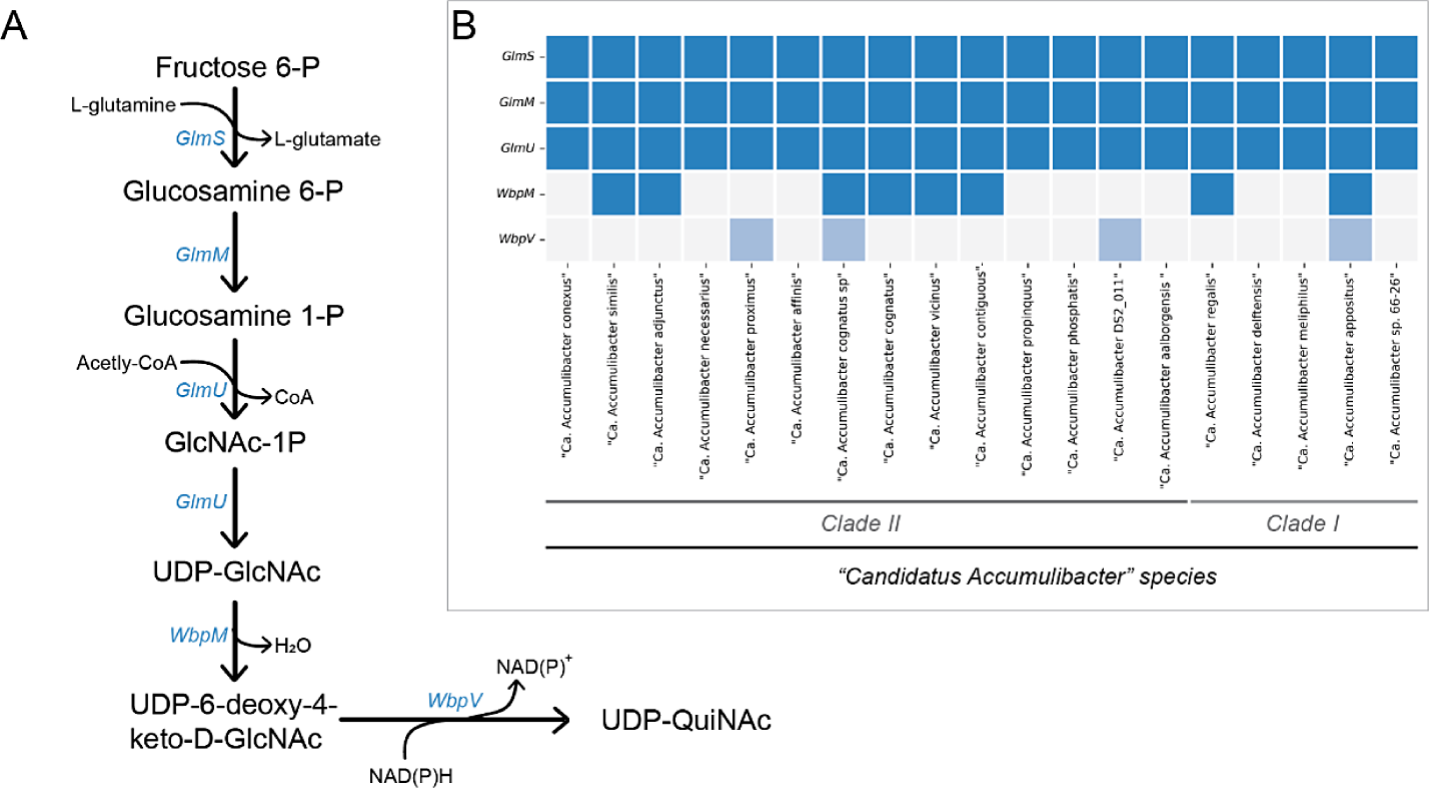
Genetic potential for the biosynthesis of QuiNAc in “*Ca.* Accumulibacter”. (A) Biosynthetic pathway of the glycan QuiNAc in bacteria indicating the genes encoding each reaction step enzyme. (B) Presence (filled with blue) or absence (empty) of the genes involved in this biosynthetic pathway in multiple metagenome assembled genomes (MAGs) of “*Ca.* Accumulibacter” species. Genes with BLAST hit >40% identity but not annotated as such are filled with lighter blue.

Guided by the identification of the rare monomer QuiNAc in the EPS of our highly enriched reactors, it is interesting to investigate the genetic potential for its biosynthesis in the ″*Ca.* Accumulibacter” species. The pathway for QuiNAc synthesis identified in *Pseudomonas aureginosa* (also present in *Rhizobium elti* and *Bacilus cereus*) is shown in Figure 3A. The first steps involve the biochemical conversions from Fructose-6-Phosphate (a glycolytic intermediate) toward UDP-GlcNAc catalyzed by the enzymes coded by *GlmS, GlmM* and *GlmU*. Next, UDP-GlcNAc is dehydrated and further oxidized by two distinct enzymes (coded by *wbpM* and *wbpV,* respectively) to generate UDP-QuiNAc. Analysis of “*Ca.* Accumulibacter” MAGs indicated that all assessed species harvested the complete gene set for synthesis up to UDP-GlcNAc (Figure 3B). For the further conversion of this sugar toward UDP-QuiNAc, several MAGs contained the *wbpM* gene, but none of the MAGs were annotated to harvest *wbpV*. Nevertheless, several MAGs contained coding sequences that were matched to *wbpV* with over 40% identity but had been annotated only as “SDR family oxidoreductases” (Figure 3 and Supporting Information). These could represent genes carrying the function of *wbpV* and thus represent the potential for QuiNAc synthesis in *“Ca.* Accumulibacter” species.

### Glycoprotein Analysis with Lectin Microarrays

Glycans include both free carbohydrates and glycoconjugates (glycoproteins and glycolipids). Since protein glycosylation is a key post-translational modification to proteins, the possible presence of glycoproteins in the EPS was studied. A lectin microarray was used to analyze the protein glycosylation within the EPS of “*Ca.* Accumulibacter”. In this assay, proteins in the extracted EPS were initially labeled with Cy3. If a protein was glycosylated, the glycan part would bind to the specific lectin present on the array and emit a fluorescent signal due to the presence of Cy3 in the protein part. Therefore, from this paper, it is possible to evaluate the presence of glycoproteins and identify the glycan profile based on the lectin specificity. In brief, a fluorescent signal signifies two things: first, the attached proteins are glycoproteins, and second, their glycan profile matches the pattern recognized by the lectin.

**Figure 4:**
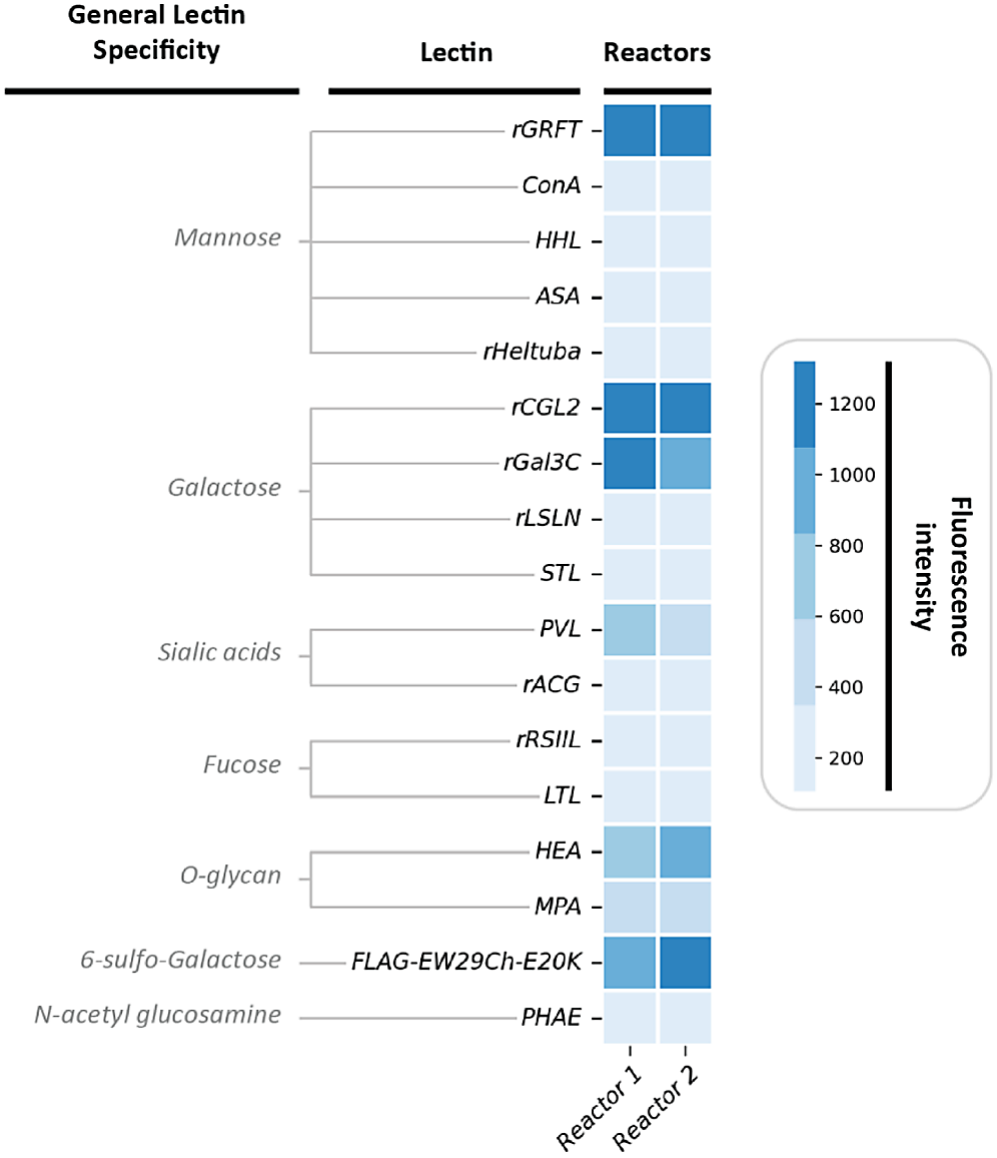
Lectin microarray profile indicating the fluorescence intensity for binding of glycoproteins in the EPS to each individual lectin. The broad specificity of each lectin is shown, and more specific structural specificity is indicated in Supporting Information.

Among the 96 lectins tested, 63 and 52 emitted a detectable fluorescent signal for the extracted EPS from reactors 1 and 2, respectively. To focus on the strongest signals, a filter (fluorescence intensity >200) was applied, sorting out 17 lectins that bound significantly to the EPS (Figure 4). It was found that the fluorescence intensity profiles were similar for both reactors 1 and 2. Notably, lectins binding glycans containing specific sugar monomers such as *rGRFT* (*mannose* containing glycans), *rCGL2* and *rGal3C* (*galactose* containing glycans), along with *PVL* (Sialic acids containing glycans), *HEA* (O-glycans) and *FLAG-EW29Ch-E200 K (6-sulfo-galactose glycan),* exhibited the highest fluorescence in both cases.

### Potential for Protein Glycosylation in “*Ca.* Accumulibacter”

**Figure 5:**
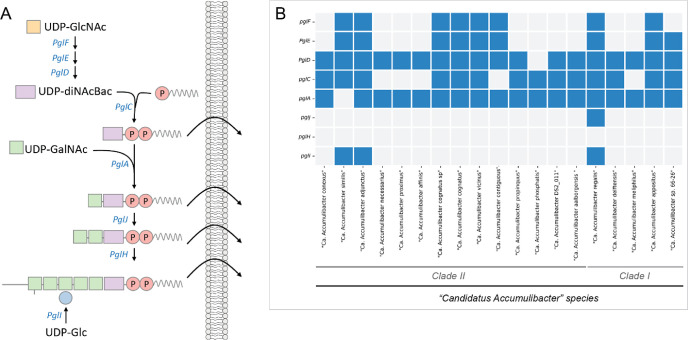
(A) Protein glycosylation mechanism present in *C. jejuni* (figure adapted from^19^). (B) Presence (filled) or absence (empty) of the genes involved in this biosynthetic pathway in multiple metagenome assembled genomes (MAGs) of “*Ca.* Accumulibacter” species.

The lectin microarray results revealed a diverse array of glycoprotein structures in the EPS of both reactors. Glycoproteins assembly typically involves the transfer of an oligosaccharide from a lipid-oligosaccharide to a protein, and the diversity stems from variations in the oligosaccharide assembly. To investigate the genetic potential responsible for lipid-oligosaccharide assembly, MAGs of “*Ca.* Accumulibacter” were compared to the well-described lipid-oligosaccharide assembly system of *Campylobacter jejuni* (Figure 5A). The analysis of gene presence and absence in diverse species of “*Ca.* Accumulibacter” revealed significant variations in the assembly system for oligosaccharides linked to glycoprotein synthesis. While some species exhibit 2 or 3 related genes, others possess near-complete systems akin to *C. jejuni* such as “*Ca.* Accumulibacter regalis” (Figure 5B). These differences imply potential species-related diversity in glycoprotein structures, as evidenced by the wide array of glycoprotein structures observed in the lectin microarrays.

## Discussion

In this research, we operated two lab-scale reactors with conditions to enrich for “*Ca.* Accumulibacter” to allow a deep understanding of the glycans and associated macromolecules produced in the EPS by members of these species. We obtained two highly enriched reactors (16S rRNA resulted in ∼70% “*Ca.* Accumulibacter” for both reactors −FISH indicated ∼95% of the biovolume) with remarkable similarities in their reactor performance and more importantly in the EPS glycans and glycoprotein profiles.

### Identification of Previously Undescribed Sugar Monomers in the EPS of “*Ca.* Accumulibacter” Enrichment

Bacteria coat themselves with a dense array of cell envelope glycans that enhance bacterial fitness and promote survival.^38^ Within a microbial aggregate, this sweet coat may end up as a component of the EPS. Additionally, glycans are specifically produced within the extracellular space. As bacterial glycans play a critical role in cell–cell and cell-environment interaction, it is significantly important to study the glycan profile of “*Ca.* Accumulibacter”, which is one of the dominant microorganisms in EBPR systems. In the current research, lab-scale reactors and various analytical methods were used to conduct this study. GC-MS analysis revealed the presence of novel glycans previously undocumented in EPS from “*Ca.* Accumulibacter”: i.e., QuiNAc and 2-OMe-Rha.

QuiNAc has been reported in bacterial species of *Pseudomonas* and *Rhizobium* associated with lipopolysaccharides (LPS)^39^ yet its function is not yet fully understood. QuiNAc-deficient mutants of *R. elti*, for example, exhibit LPS with significant reduction in the O-antigen content compared to the wild type. Such mutants fail to aggregate and colonize nodules in the roots of their legume hosts^40^ even when the O-antigen content is increased by genetic engineering.^41^ Thus, QuiNAc is proposed to serve as the bridging glycan between lipids and oligosaccharides in LPS.^42−44^ It is worth pointing out the similarities between *R. elti* and “*Ca.* Accumulibacter” since both bacterial species appear to grow as densely aggregated microcolonies. In this respect, the role and the exact location of QuiNAc in “*Ca.* Accumulibacter*”* requires further research, which may shed light on maintaining a stable population of “*Ca.* Accumulibacter*”* in the EBPR system at wastewater treatment plant.

Besides QuiNAc, 2-O-methyl-rhamnose is another uncommon sugar monomer detected in the EPS of “*Ca.* Accumulibacter”. 2-O-methyl-rhamnose has been reported on the S-layer glycoprotein glycan of *Geobacillus stearothermophilus.*(45) It has also been reported as part of the repeating unit of the lipopolysaccharide from *Thiocapsa roseopersicina*(46) and as a spore-specific constituent of *Bacillus cereus*.^47^ The role of 2-O-methyl-rhamnose is not clearly described in the literature. It was hypothesized that 2-O-methylation of the terminal rhamnose residue on the S-layer glycoprotein glycan of *G. stearothermophilus* might function as a termination signal for chain elongation (van Teeseling et al).^203^ Why it is produced by “*Ca.* Accumulibacter” enrichment and where it is located are interesting topics to be investigated.

### Glycoproteins Are Present and Highly Diverse in the EPS of “*Ca.* Accumulibacter”

Within the glycans, in addition to free polysaccharides, there are glycoconjugates such as glycoproteins and glycolipids. To further investigate the potential existence of glycoproteins and their glycan profile, a lectin microarray analysis was performed. The existence of glycoproteins with diverse glycosylation patterns were observed. Protein glycosylation has profound effects on the protein function and stability. For example, the surface layer proteins, which envelop almost all bacteria, have glycosylation patterns that significantly influence properties like water retention, surface roughness and fluidity.^48^ In environmental microorganisms such as “*Ca.* Accumulibacter”, both the presence and the strong diversity of glycoproteins in the EPS may be crucial for the functioning and assembly of the microbial community. This has significant implications for comprehending the role of EPS proteins since their functionality and structure can be fundamentally different depending on the type and diversity of the associated glycans.^49^

Typically, approaches for studying glycoproteins in environmental samples involve identifying individual glycan structures and further characterizing the proteins with mass spectrometry.^21,50,51^ Recently, ref^14^ introduced a systematic glycoproteomics method, revealing a wide array of glycoproteins in an enrichment culture of anaerobic ammonium-oxidizing bacteria, aligning with our findings for similar environmental bacteria. While the described glycoproteomics approach effectively identifies specific proteins and glycan compositions, lectin microarrays, such as the method applied in this study, offer a high throughput examination of the glycans on the protein surfaces, enabling a broader screening of a possible protein glycosylation pattern. Combining both approaches can provide a comprehensive understanding of glycoproteins, bridging the gap between structural characterization and functional implications.

### In EPS Research, Identifying Novel Glycans and Glycoconjugates Needs a Bottom-Up Approach

Bacteria produce a tremendous variety of unusual sugars and sugar linkages as well as modifications of sugars. The study of bacterial glycans is further complicated by their enormous structural diversity. In comparison, mammalian cells construct their cell surface glycans using only nine monosaccharide building blocks, plants use 12 monosaccharides, whereas >700 monosaccharides have been found in bacterial glycans.^38^

Moreover, unlike DNA replication or protein translation, glycan biosynthesis is not directed by a preexisting template molecule. Instead, the production of glycans is decided by a few factors: the biosynthetic machinery, the available nucleotide sugars (which serve as monosaccharide donors), and signals from the intracellular and extracellular environment. Thus, the presence of glycans is dynamic and is influenced by both genetic and environmental factors.^52^ Therefore, if the factors influencing glycan production and the remarkable variety of monosaccharides that can be produced by bacteria are added up, it is tremendously challenging to study the glycan composition in EPS.

Currently, *top-down* approaches are widely used. They predict the glycans composition in the EPS based on metagenomes,^13^ resulting in *theoretical* polymeric substances that require experimental validation.^53^ In addition, bioinformatic approaches based on DNA sequence are limited to only discovering biosynthetic pathways that have been very well described (e.g., cellulose biosynthesis,^54^ shadowing the identification of unknown biomolecules and new glycans. As an example, in this research, the sugar monomer QuiNAc was detected in highly enriched “*Ca.* Accumulibacter” cultures, while its complete biosynthetic pathway could not be obtained in the available high quality genomes of *“Ca.* Accumulibacter”. This indicates that with the top-down approach, the existence of QuiNAc can hardly be predicted.

Findings of the current research, together with those of ref^14^, highlight the huge diversity encountered in the glycans in the EPS. Homology modeling of enzymes involved in carbohydrate synthesis and transfer rarely provides information on the type of monosaccharide involved in the process^55^ which further hinders a complete description from metagenome information only. We identified that the genomes of “*Ca.* Accumulibacter” species harbor different sets of genes in the described system for oligosaccharide-lipid assembly, showcasing that even on a genus level there is a high potential for varying glycan compositions. Due to the special property of glycan synthesis, it is significantly necessary to use more *bottom-up* approaches for the chemical description of EPS components, which could guide further genetic analysis and generalizations.

## Conclusions

– Novel glycans containing QuiNAc and 2-OMe-Rha were identified for the first time in the EPS of “*Ca.* Accumulibacter” enrichments.
– Glycoproteins in the EPS from “*Ca.* Accumulibacter” are present and exhibit a high variation in the glycan structures that make them.
– The complexity in the EPS of environmental bacteria hinder the *top-down* approaches to only discover well-known polymeric substances. More *bottom-up* research is required to fill the knowledge gap that is required for genomic modeling approaches to understanding EPS of environmental bacteria.

## Supporting Information Available

Experimental methods, gene analysis data, FISH and EPS chromatograms (PDF)

## References

[ref1] Beun J.; Hendriks A.; Van Loosdrecht M.; Morgenroth E.; Wilderer P.; Heijnen J. Aerobic granulation in a sequencing batch reactor. Water Res. 1999, 33 (10), 2283–2290. 10.1016/S0043-1354(98)00463-1.

[ref2] Seviour R.; Nielsen P. H. Microbial ecology of activated sludge; IWA publishing, 2010.

[ref3] Flemming H.-C.; Wingender J. The biofilm matrix. Nat. Rev. Microbiol. 2010, 8 (9), 623–633. 10.1038/nrmicro2415. 20676145

[ref4] Kleikamp H. B.; Grouzdev D.; Schaasberg P.; van Valderen R.; van der Zwaan R.; van de Wijgaart R.; Lin Y.; Abbas B.; Pronk M.; van Loosdrecht M. C. Comparative metaproteomics demonstrates different views on the complex granular sludge microbiome bioRxiv 2022 10.1101/2022.03.07.483319. 37866247

[ref5] Fro̷lund B.; Palmgren R.; Keiding K.; Nielsen P. H. Extraction of extracellular polymers from activated sludge using a cation exchange resin. Water Res. 1996, 30 (8), 1749–1758. 10.1016/0043-1354(95)00323-1.

[ref6] Whitton B. A. Ecology of cyanobacteria II: Their diversity in space and time; Springer Science & Business Media, 2012.

[ref7] Pinchuk G. E.; Ammons C.; Culley D. E.; Li S.-M. W.; McLean J. S.; Romine M. F.; Nealson K. H.; Fredrickson J. K.; Beliaev A. S. Utilization of DNA as a sole source of phosphorus, carbon, and energy by Shewanella spp.: Ecological and physiological implications for dissimilatory metal reduction. Appl. Environ. Microbiol. 2008, 74 (4), 1198–1208. 10.1128/AEM.02026-07. 18156329 PMC2258558

[ref8] Flemming H.-C.; van Hullebusch E. D.; Neu T. R.; Nielsen P. H.; Seviour T.; Stoodley P.; Wingender J.; Wuertz S. The biofilm matrix: Multitasking in a shared space. Nat. Rev. Microbiol. 2023, 21 (2), 70–86. 10.1038/s41579-022-00791-0. 36127518

[ref9] Liu Y.; Fang H. H. P. Influences of extracellular polymeric substances (EPS) on flocculation, settling, and dewatering of activated sludge. Crit. Rev. Environ. Sci. Technol. 2003, 33, 237–273. 10.1080/10643380390814479.

[ref10] Felz S.; Al-Zuhairy S.; Aarstad O. A.; van Loosdrecht M. C.; Lin Y. M. Extraction of structural extracellular polymeric substances from aerobic granular sludge. JoVE 2016, (115), e54534 10.3791/54534. PMC5092066 27768085

[ref11] Felz S.; Vermeulen P.; van Loosdrecht M. C.; Lin Y. M. Chemical characterization methods for the analysis of structural extracellular polymeric substances (EPS). Water Res. 2019, 157, 201–208. 10.1016/j.watres.2019.03.068. 30953855

[ref12] Lens P.; O’Flaherty V.; Moran A.; Stoodley P.; Mahony T. Biofilms in medicine, industry and environmental biotechnology; IWA publishing, 2003.

[ref13] Dueholm M. K. D.; Besteman M.; Zeuner E. J.; Riisgaard-Jensen M.; Nielsen M. E.; Vestergaard S. Z.; Heidelbach S.; Bekker N. S.; Nielsen P. H. Genetic potential for exopolysaccharide synthesis in activated sludge bacteria uncovered by genome-resolved metagenomics. Water Res. 2023, 229, 119485 10.1016/j.watres.2022.119485. 36538841

[ref14] Pabst M.; Grouzdev D. S.; Lawson C. E.; Kleikamp H. B.; de Ram C.; Louwen R.; Lin Y. M.; Lücker S.; van Loosdrecht M. C.; Laureni M. A general approach to explore prokaryotic protein glycosylation reveals the unique surface layer modulation of an anammox bacterium. ISME J 2022, 16 (2), 346–357. 10.1038/s41396-021-01073-y. 34341504 PMC8776859

[ref15] Santander J.; Martin T.; Loh A.; Pohlenz C.; Gatlin Iii D. M.; Curtiss Iii R. Mechanisms of intrinsic resistance to antimicrobial peptides of Edwardsiella ictaluri and its influence on fish gut inflammation and virulence. Microbiology 2013, 159 (Pt 7), 1471 10.1099/mic.0.066639-0. 23676433 PMC4085987

[ref16] Peña M. J.; Tuomivaara S. T.; Urbanowicz B. R.; O’Neill M. A.; York W. S. Methods for structural characterization of the products of cellulose-and xyloglucan-hydrolyzing enzymes. Methods Enzymol. 2012, 510, 121–139. 10.1016/B978-0-12-415931-0.00007-0. 22608724

[ref17] Tateno H.; Toyota M.; Saito S.; Onuma Y.; Ito Y.; Hiemori K.; Fukumura M.; Matsushima A.; Nakanishi M.; Ohnuma K.; et al. Glycome diagnosis of human induced pluripotent stem cells using lectin microarray. J. Biol. Chem. 2011, 286 (23), 20345–20353. 10.1074/jbc.M111.231274. 21471226 PMC3121447

[ref18] Mescher M. F.; Strominger J. L. Purification and characterization of a prokaryotic glycoprotein from the cell envelope of Halobacterium salinarium. J. Biol. Chem. 1976, 251 (7), 2005–2014. 10.1016/S0021-9258(17)33647-5. 1270419

[ref19] Nothaft H.; Szymanski C. M. Protein glycosylation in bacteria: Sweeter than ever. Nat. Rev. Microbiol. 2010, 8 (11), 765–778. 10.1038/nrmicro2383. 20948550

[ref20] Formosa-Dague C.; Castelain M.; Martin-Yken H.; Dunker K.; Dague E.; Sletmoen M. The role of glycans in bacterial adhesion to mucosal surfaces: How can single-molecule techniques advance our understanding?. Microorganisms 2018, 6 (2), 39 10.3390/microorganisms6020039. 29734645 PMC6027152

[ref21] Boleij M.; Pabst M.; Neu T. R.; Van Loosdrecht M. C.; Lin Y. Identification of glycoproteins isolated from extracellular polymeric substances of full-scale anammox granular sludge. Environ. Sci. Technol. 2018, 52 (22), 13127–13135. 10.1021/acs.est.8b03180. 30335377 PMC6256349

[ref22] Szymanski C. M.; Yao R.; Ewing C. P.; Trust T. J.; Guerry P. Evidence for a system of general protein glycosylation in Campylobacter jejuni. Mol. Microbiol. 1999, 32 (5), 1022–1030. 10.1046/j.1365-2958.1999.01415.x. 10361304

[ref23] Wacker M.; Linton D.; Hitchen P. G.; Nita-Lazar M.; Haslam S. M.; North S. J.; Panico M.; Morris H. R.; Dell A.; Wren B. W.; et al. N-linked glycosylation in Campylobacter jejuni and its functional transfer into E. coli. Science 2002, 298 (5599), 1790–1793. 10.1126/science.298.5599.1790. 12459590

[ref24] Gross J.; Grass S.; Davis A. E.; Gilmore-Erdmann P.; Townsend R. R.; Geme J. W. S. The Haemophilus influenzae HMW1 adhesin is a glycoprotein with an unusual N-linked carbohydrate modification. J. Biol. Chem. 2008, 283 (38), 26010–26015. 10.1074/jbc.M801819200. 18621734 PMC3258857

[ref200] Guedes da Silva L.; Olavarria Gamez K.; Castro Gomes J.; Akkermans K.; Welles L.; Abbas B.; van Loosdrecht M. C. M.; Wahl S. A. Revealing the Metabolic Flexibility of “Candidatus Accumulibacter phosphatis” through Redox Cofactor Analysis and Metabolic Network Modeling. Applied Environmental Microbiology 2020, 86, e00808-20 10.1128/AEM.00808-20. 33008826 PMC7688218

[ref201] Smolders G. J. F.; van der Meij J.; van Loosdrecht M. C. M.; Heijnen J. J. Model of the anaerobic metabolism of the biological phosphorus removal process: stoichiometry and pH influence. Biotechnology and bioengineering 1994, 43, 461–470. 10.1002/bit.260430605. 18615742

[ref25] Caporaso J. G.; Kuczynski J.; Stombaugh J.; Bittinger K.; Bushman F. D.; Costello E. K.; Fierer N.; Peña A. G.; Goodrich J. K.; Gordon J. I.; et al. QIIME allows analysis of high-throughput community sequencing data. Nat. Methods 2010, 7 (5), 335–336. 10.1038/nmeth.f.303. 20383131 PMC3156573

[ref26] Edgar R. C.; Haas B. J.; Clemente J. C.; Quince C.; Knight R. UCHIME improves sensitivity and speed of chimera detection. Bioinformatics 2011, 27 (16), 2194–2200. 10.1093/bioinformatics/btr381. 21700674 PMC3150044

[ref27] Edgar R. C. UPARSE: Highly accurate OTU sequences from microbial amplicon reads. Nat. Methods 2013, 10 (10), 996–998. 10.1038/nmeth.2604. 23955772

[ref28] Quast C.; Pruesse E.; Yilmaz P.; Gerken J.; Schweer T.; Yarza P.; Peplies J.; Glöckner F. O. The SILVA ribosomal RNA gene database project: Improved data processing and web-based tools. Nucleic Acids Res. 2012, 41 (D1), D590–D596. 10.1093/nar/gks1219. 23193283 PMC3531112

[ref29] Winkler M.-K.; Bassin J.; Kleerebezem R.; De Bruin L.; Van den Brand T.; Van Loosdrecht M. Selective sludge removal in a segregated aerobic granular biomass system as a strategy to control PAO–GAO competition at high temperatures. Water Res. 2011, 45 (11), 3291–3299. 10.1016/j.watres.2011.03.024. 21513967

[ref30] Amann R. I.; Binder B. J.; Olson R. J.; Chisholm S. W.; Devereux R.; Stahl D. Combination of 16S rRNA-targeted oligonucleotide probes with flow cytometry for analyzing mixed microbial populations. Appl. Environ. Microbiol. 1990, 56 (6), 1919–1925. 10.1128/aem.56.6.1919-1925.1990. 2200342 PMC184531

[ref31] Daims H.; Brühl A.; Amann R.; Schleifer K.-H.; Wagner M. The domain-specific probe EUB338 is insufficient for the detection of all Bacteria: Development and evaluation of a more comprehensive probe set. Syst. Appl. Microbiol. 1999, 22 (3), 434–444. 10.1016/S0723-2020(99)80053-8. 10553296

[ref32] Crocetti G. R.; Hugenholtz P.; Bond P. L.; Schuler A.; Keller J. R.; Jenkins D.; Blackall L. L. Identification of polyphosphate-accumulating organisms and design of 16S rRNA-directed probes for their detection and quantitation. Appl. Environ. Microbiol. 2000, 66 (3), 1175–1182. 10.1128/AEM.66.3.1175-1182.2000. 10698788 PMC91959

[ref33] Smith P. E.; Krohn R. I.; Hermanson G.; Mallia A.; Gartner F.; Provenzano M.; Fujimoto E.; Goeke N.; Olson B.; Klenk D. Measurement of protein using bicinchoninic acid. Anal. Biochem. 1985, 150 (1), 76–85. 10.1016/0003-2697(85)90442-7. 3843705

[ref34] DuBois M.; Gilles K. A.; Hamilton J. K.; Rebers P. T.; Smith F. Colorimetric method for determination of sugars and related substances. Anal. Chem. 1956, 28 (3), 350–356. 10.1021/ac60111a017.

[ref35] Merkle R. K.; Poppe I. [1] Carbohydrate composition analysis of glycoconjugates by gas-liquid chromatography/mass spectrometry. Methods Enzymol. 1994, 230, 1–15. 10.1016/0076-6879(94)30003-8. 8139491

[ref36] Páez-Watson T.; van Loosdrecht M. C.; Wahl S. A. From metagenomes to metabolism: Systematically assessing the metabolic flux feasibilities for “Candidatus Accumulibacter” species during anaerobic substrate uptake. Water Res. 2024, 250, 121028 10.1016/j.watres.2023.121028. 38128304

[ref37] Welles L.; Tian W.; Saad S.; Abbas B.; Lopez-Vazquez C.; Hooijmans C.; Van Loosdrecht M.; Brdjanovic D. Accumulibacter clades Type I and II performing kinetically different glycogen-accumulating organisms metabolisms for anaerobic substrate uptake. Water Res. 2015, 83, 354–366. 10.1016/j.watres.2015.06.045. 26189167

[ref38] Barrett K.; Dube D. H. Chemical Tools to Study Bacterial Glycans: A Tale from Discovery of Glycoproteins to Disruption of Their Function. Isr. J. Chem. 2023, 63 (1–2), e202200050 10.1002/ijch.202200050. 37324574 PMC10266715

[ref39] Li T.; Simonds L.; Kovrigin E. L.; Noel K. D. In vitro biosynthesis and chemical identification of UDP-N-acetyl-d-quinovosamine (UDP-d-QuiNAc). J. Biol. Chem. 2014, 289 (26), 18110–18120. 10.1074/jbc.M114.555862. 24817117 PMC4140256

[ref40] Cava J. R.; Elias P. M.; Turowski D. A.; Noel K. D. Rhizobium leguminosarum CFN42 genetic regions encoding lipopolysaccharide structures essential for complete nodule development on bean plants. J. Bacteriol. 1989, 171 (1), 8–15. 10.1128/jb.171.1.8-15.1989. 2644215 PMC209546

[ref41] Noel K. D.; Forsberg L. S.; Carlson R. W. Varying the abundance of O antigen in Rhizobium etli and its effect on symbiosis with Phaseolus vulgaris. J. Bacteriol. 2000, 182 (19), 5317–5324. 10.1128/JB.182.19.5317-5324.2000. 10986232 PMC110972

[ref42] Forsberg L. S.; Bhat U. R.; Carlson R. W. Structural characterization of the o-antigenic polysaccharide of the lipopolysaccharide from Rhizobium etli strain CE3: A unique O-acetylated glycan of discrete size, containing 3-O-methyl-6-deoxy-L-talose and 2, 3, 4-tri-O-methyl-L-fucose. J. Biol. Chem. 2000, 275 (25), 18851–18863. 10.1074/jbc.M001090200. 10858446

[ref43] Ojeda K. J.; Simonds L.; Noel K. D. Roles of predicted glycosyltransferases in the biosynthesis of the Rhizobium etli CE3 O antigen. J. Bacteriol. 2013, 195 (9), 1949–1958. 10.1128/JB.02080-12. 23435981 PMC3624598

[ref44] Li T.; Noel K. D. Synthesis of N-acetyl-d-quinovosamine in Rhizobium etli CE3 is completed after its 4-keto-precursor is linked to a carrier lipid. Microbiology 2017, 163 (12), 1890 10.1099/mic.0.000576. 29165235 PMC5845739

[ref45] Schäffer C.; Wugeditsch T.; Kählig H.; Scheberl A.; Zayni S.; Messner P. The surface layer (S-layer) glycoprotein of Geobacillus stearothermophilus NRS 2004/3a: Analysis of its glycosylation. J. Biol. Chem. 2002, 277 (8), 6230–6239. 10.1074/jbc.M108873200. 11741945

[ref46] Hurlbert R. E.; Weckesser J.; Tharanathan R. N.; Mayer H. Isolation and characterization of the lipopolysaccharide of Thiocapsa roseopersicina. Eur. J. Biochem. 1978, 90 (2), 241–246. 10.1111/j.1432-1033.1978.tb12596.x. 710428

[ref47] Fox A.; Black G. E.; Fox K.; Rostovtseva S. Determination of carbohydrate profiles of Bacillus anthracis and Bacillus cereus including identification of O-methyl methylpentoses by using gas chromatography-mass spectrometry. J. Clin. Microbiol. 1993, 31 (4), 887–894. 10.1128/jcm.31.4.887-894.1993. 8463400 PMC263582

[ref203] van Teeseling M. C. F.; Maresch D.; Rath C. B.; Figl R.; Altmann F.; Jetten M. S. M.; Messner P.; Schaffer C.; van Niftrik L. The S-layer protein of the anammox bacterium Kuenenia stuttgartiensis is heavily O-glycosylated. Frontiers in microbiology 2016, 7, 1721 10.3389/fmicb.2016.01721. 27847504 PMC5088730

[ref48] Schuster B.; Sleytr U. B. Relevance of glycosylation of S-layer proteins for cell surface properties. Acta Biomater. 2015, 19, 149–157. 10.1016/j.actbio.2015.03.020. 25818946 PMC4414373

[ref49] Lepenies B.; Seeberger P. H. Simply better glycoproteins. Nat. Biotechnol. 2014, 32 (5), 443–445. 10.1038/nbt.2893. 24811516

[ref50] Fulton K. M.; Li J.; Tomas J. M.; Smith J. C.; Twine S. M. Characterizing bacterial glycoproteins with LC-MS. Expert Rev. Proteomics 2018, 15 (3), 203–216. 10.1080/14789450.2018.1435276. 29400572

[ref51] Van Teeseling M. C. F.; Maresch D.; Rath C. B.; Figl R.; Altmann F.; Jetten M. S. M.; Messner P.; Schäffer C.; Van Niftrik L. The S-layer protein of the anammox bacterium Kuenenia stuttgartiensis is heavily O-glycosylated. Front. Microbiol. 2016, 7, 1721 10.3389/fmicb.2016.01721. 27847504 PMC5088730

[ref52] Chen L. M.; de Bruin S.; Pronk M.; Sousa D. Z.; van Loosdrecht M. C.; Lin Y. Sialylation and Sulfation of Anionic Glycoconjugates Are Common in the Extracellular Polymeric Substances of Both Aerobic and Anaerobic Granular Sludges. Environ. Sci. Technol. 2023, 57, 13217–13225. 10.1021/acs.est.2c09586. 37604486 PMC10483923

[ref53] Seviour T.; Derlon N.; Dueholm M. S.; Flemming H.-C.; Girbal-Neuhauser E.; Horn H.; Kjelleberg S.; van Loosdrecht M. C. M.; Lotti T.; Malpei M. F.; et al. Extracellular polymeric substances of biofilms: Suffering from an identity crisis. Water Res. 2019, 151, 1–7. 10.1016/j.watres.2018.11.020. 30557778

[ref54] McNamara J. T.; Morgan J. L.; Zimmer J. A molecular description of cellulose biosynthesis. Annu. Rev. Biochem. 2015, 84, 895–921. 10.1146/annurev-biochem-060614-033930. 26034894 PMC4710354

[ref55] Szymanski C. M. Bacterial glycosylation, it’s complicated. Front. Mol. Biosci. 2022, 9, 1015771 10.3389/fmolb.2022.1015771. 36250013 PMC9561416

