# Supplementary material for: Sweet Secrets: Exploring Novel Glycans and Glycoconjugates in the Extracellular Polymeric Substances of “Candidatus Accumulibacter”
Source: ACS ES T Water. 2024 Jul 12;4(8):3391–9. doi: 10.1021/acsestwater.4c00247 (PMC11320575; doi:10.1021/acsestwater.4c00247)
Supplement: Supplementary file 1 — ew4c00247_si_001.pdf [file ew4c00247_si_001.pdf]

Supplemental material

Sweet Secrets: Exploring Novel Glycans and Glycoconjugates in the Extracellular Polymeric Substances of “*Candidatus* Accumulibacter”

Timothy Páez-Watson[1], Sergio Tomás-Martínez[1], Roeland de Wit[1], Sunanda Keisham[2], Hiroaki Tateno[2], Mark C.M. van Loosdrecht[1], Yuemei Lin[1]*

[1] Department of Biotechnology, Delft University of Technology. Van der Maasweg 9,2629 HZ, Delft, The Netherlands

[2] Cellular and Molecular Biotechnology Research Institute, National Institute of Advanced Industrial Science and Technology (AIST), Central 6, 1-1-1 Higashi, Tsukuba, Ibaraki 305-8566, Japan

Corresponding Author: Timothy Páez-Watson

Address: Department of Biotechnology, Delft University of Technology. Van der Maasweg 9,2629 HZ, Delft, The Netherlands

E-Mail: T.W.PaezWatson@tudelft.nl

Genomes from *“Candidatus* Accumulibacter”

Table S1. *Species of “Candidatus Accumulibacter” considered in this study. All sequences were downloaded from the European Nucleotide Archive (ENA) as WGS-EMBL.*

Specie	Accession number	PAOs clade
Candidatus Accumulibacter conexus	GCA_017592775.1	Clade II
Candidatus Accumulibacter similis	GCA_003332265.1	
Candidatus Accumulibacter adjunctus	GCA_000585015.1	
Candidatus Accumulibacter necessarius	GCA_017302435.1	
Candidatus Accumulibacter proximus	GCA_016709675.1	
Candidatus Accumulibacter affinis	GCA_016713625.1	
Candidatus Accumulibacter cognatus sp	GCA_000584975.2	
Candidatus Accumulibacter cognatus	GCA_005889575.1	
Candidatus Accumulibacter vicinus	GCA_000584955.2	
Candidatus Accumulibacter contiguus	GCA_012940005.1	
Candidatus Accumulibacter propinquus	GCA_017302555.1	
Candidatus Accumulibacter phosphatis	GCA_017592745.1	
Candidatus Accumulibacter DS2_011	GCA_013823155.1	
Candidatus Accumulibacter aalborgensis	GCA_900089955.1	
Candidatus Accumulibacter regalis	GCA_017592785.1	Clade I
Candidatus Accumulibacter delftensis	GCA_012939955.1	
Candidatus Accumulibacter meliphilus	GCA_003332265.1	
Candidatus Accumulibacter appositus	GCA_000585055.1	
Candidatus Accumulibacter sp. 66-26	GCA_001897745.1	

Genes and protein sequences used for BLAST

Table S2. *Genes and Uniprot codes of the protein sequences downloaded for performing BLAST on species of "Candidatus Accumulibacter".*

Protein	Uniprot ID
GlmS	P17169
GlmM	P31120
GlmU	P0ACC7
wbpM	Q8GGB3
wbpV	AAF23991.1
pglF	Q0P9D4
pglE	Q0P9D3
pglD	Q0P9D1
pglC	Q0P9D0
pglA	Q0P9C9
pglJ	Q0P9C7
pglH	Q0P9C5
pglI	Q0P9C6

‘Oxidoreductases’ identified with > 40 % sequence identity to wbpV

Table S3. *BLAST results from the alignment of “Ca. Accumulibacter” MAGs to wpbV proteins.*

Species	Identity	E-value	protein name	location
Candidatus Accumulibacter regalis	43,81	1,43E-84	dTDP-glucose 4,6-dehydratase	[109721:110687](-)
Candidatus Accumulibacter regalis	43,81	1,43E-84	dTDP-glucose 4,6-dehydratase	[109721:110687](-)
Candidatus Accumulibacter regalis	43,81	1,43E-84	dTDP-glucose 4,6-dehydratase	[109721:110687](-)
Candidatus Accumulibacter regalis	43,81	1,43E-84	dTDP-glucose 4,6-dehydratase	[109721:110687](-)
Candidatus Accumulibacter sp. 66-26	56,55	1,22E-119	hypothetical protein	[196637:197585](+)
Candidatus Accumulibacter sp. 66-26	56,55	1,22E-119	hypothetical protein	[196637:197585](+)
Candidatus Accumulibacter sp. 66-26	56,55	1,22E-119	hypothetical protein	[196637:197585](+)
Candidatus Accumulibacter sp. 66-26	56,55	1,22E-119	hypothetical protein	[196637:197585](+)
Candidatus Accumulibacter DS2_011	57,188	7,83E-121	NAD-dependent dehydratase	[188793:189741](+)
Candidatus Accumulibacter DS2_011	57,188	7,83E-121	NAD-dependent dehydratase	[188793:189741](+)

Candidatus Accumulibacter DS2_011	57,188	7,83E-121	NAD-dependent dehydratase	[188793:189741](+)
Candidatus Accumulibacter DS2_011	57,188	7,83E-121	NAD-dependent dehydratase	[188793:189741](+)
Candidatus Accumulibacter regalis	47,771	3,44E-91	**SDR family oxidoreductase**	[80260:81235](+)
Candidatus Accumulibacter regalis	47,771	3,44E-91	**SDR family oxidoreductase**	[80260:81235](+)
Candidatus Accumulibacter regalis	47,771	3,44E-91	**SDR family oxidoreductase**	[80260:81235](+)
Candidatus Accumulibacter regalis	47,771	3,44E-91	**SDR family oxidoreductase**	[80260:81235](+)
Candidatus Accumulibacter phosphatis	60,828	5,06E-116	**SDR family oxidoreductase**	[25017:25983](+)
Candidatus Accumulibacter phosphatis	60,828	5,06E-116	**SDR family oxidoreductase**	[25017:25983](+)
Candidatus Accumulibacter propinquus	52,548	4,64E-105	NAD-dependent epimerase/dehydratase family protein	[27937:28939](+)
Candidatus Accumulibacter propinquus	52,548	4,64E-105	NAD-dependent epimerase/dehydratase family protein	[27937:28939](+)
Candidatus Accumulibacter phosphatis	60,828	4,89E-116	**SDR family oxidoreductase**	[130936:131902](+)
Candidatus Accumulibacter phosphatis	60,828	4,89E-116	**SDR family oxidoreductase**	[130936:131902](+)

Candidatus Accumulibacter regalis	47,771	3,4E-91	SDR family oxidoreductase	[2280:3255](-)
Candidatus Accumulibacter regalis	47,771	3,4E-91	SDR family oxidoreductase	[2280:3255](-)
Candidatus Accumulibacter regalis	47,771	3,4E-91	SDR family oxidoreductase	[2280:3255](-)
Candidatus Accumulibacter regalis	47,771	3,4E-91	SDR family oxidoreductase	[2280:3255](-)

Glycosyl composition of extracted EPS

Table S4. *Glycosyl composition of the extracted EPS as relative mole abundance from the total amount of carbohydrate monomers determined by GC-MS. Carbohydrate monomers detected: glucose (Glc), Rhamnose (Rha), Mannose (Man), Galactose (Gal), Ribose (Rib,) N-Acetylglucosamine (GlcNAc), N-Acetylquinovosamine (QuiNAc) and 2-O-Methylrhamnose (2-OMe-Rha).*

Glycosyl Residue	Abbreviation	Reactor 1	Reactor 2
Mannose	Man	0.6	0.7
2-O-Mthylrhamnose	2-OMe-Rha	0.7	0.8
Ribose	Rib	1.7	1.7
N-Acetylquinovosamine	QuiNAc	2.1	2.3
Galactose	Gal	2.7	2.0
Rhamnose	Rha	3.5	3.5
N-Acetylglucosamine	GlcNAc	4.2	2.4
Glucose	Glc	84.6	86.6

Lectin array and specificity

Table S5. *Lectin microarray profile indicating the fluorescence intensity for binding of glycoproteins in the EPS to each individual lectin with its rough glycan specificity.*

Lectin ID	Rough glycan specificity	Reactor 1 (fluorescence intensity)		Reactor 2 (fluorescence intensity)	
LFA	Sia	0	0	0	0
WGA	(GlcNAc)n, polySia	0	0	0	0
rGRFT	Man	1311,95	1327,48	1100,14	1150,28
ConA	M3, Manα1-2Manα1-3(Manα1-6)Man, GlcNAcβ1-2Manα1-3(Manα1-6)Man	286,21	300,98	280,18	260,29
HHL	Manα1-3Man, Manα1-6Man	231,18	220,48	278,11	299,50
ASA	Galβ1-4GlcNAcβ1-2Man	105,48	107,51	115,30	126,30
rHeltuba	Manα1-3Man	248,02	172,84	137,83	169,20
rCGL2	GalNAcα1-3Gal (A), PolyLacNAc	1233,52	1289,74	1095,00	1076,52
rGal3C	LacNAc, polylactosamine	944,84	1153,71	1164,62	1177,81
rLSLN	LacNAc, polylactosamine	167,30	200,36	242,84	233,48
STL	Polylactosamine, (GlcNAc)n	125,03	137,67	198,51	216,64
PVL	Sia, GlcNAc	575,79	582,32	655,37	603,60
MAL	α2-3Sia	14,16	16,92	25,00	27,11
MAH	α2-3Sia	42,91	45,27	48,67	52,30
ACG	α2-3Sia	0,98	1,19	12,30	14,09
rACG	α2-3Sia	187,53	210,79	299,42	288,92
rRSIIL	α1-2Fuc (H), α1-3Fuc (Lex), α1-4Fuc (Lea)	201,07	203,82	190,01	185,51
LTL	Lex, Ley	98,70	109,45	145,40	144,16
HEA	Galβ1-3GalNAc (T)	1059,90	669,30	722,80	816,98
MPA	Galβ1-3GalNAc (T), GalNAcα (Tn)	532,91	599,29	492,82	478,81
FLAG-EW29Ch-E20K	6-sulfo-Gal	1192,29	1339,31	970,11	857,48
PHAE	bisecting GlcNAc	162,42	160,87	152,85	153,37
rGal8N	α2-3Sia	0,00	0,00	0,00	0,00
SNA	α2-6Sia	0,91	0,02	12,21	15,29
SSA	α2-6Sia	62,55	52,20	63,45	22,17
TJAI	α2-6Sia	0,05	0,07	0,45	0,62

rPSL1a	α2-6Sia	59,63	77,97	77,30	56,43
ADA	α2-6Sia, Forssman, A, B	0,00	0,00	0,00	0,00
PHAL	GlcNAcβ1-6Man (Tetraantenna)	22,18	18,08	32,36	36,27
DSA	GlcNAcβ1-6Man (Tetraantenna)	6,66	6,51	24,61	28,22
TxLcI	Galactosylated N-glycans up to triantenna	67,13	65,88	79,77	90,77
ECA	βGal	0,14	0,00	3,24	3,90
RCA120	βGal	0,00	0,00	0,00	0,00
rGal7	Type1 LacNAc, chondroitin polymer	85,88	81,76	158,09	143,81
rGal9N	GalNAcα1-4Gal (A), PolyLacNAc	0,00	0,00	0,00	0,00
rGal9C	PolyLacNAc, Branched LacNAc	0,00	0,00	0,00	0,00
rC14	Branched LacNAc	5,27	2,99	21,74	23,83
rDiscoidin II	LacNAc, Galβ1-3GalNAc (T), GalNAc (Tn)	0,00	0,00	0,00	0,00
BPL	Galβ1-3GlcNAc(GalNAc), a/βGalNAc	19,62	17,27	26,98	26,64
GSLII	GlcNAcβ1-4Man	0,00	0,00	0,00	0,00
rSRL	Core1,3, agalacto N-glycan	0,10	0,40	1,14	0,85
UDA	(GlcNAc)n	0,00	0,00	0,00	0,02
PWM	(GlcNAc)n	0,00	0,00	0,00	0,00
rF17AG	GlcNAc	15,95	0,32	2,47	12,14
NPA	Manα1-3Man	0,00	0,00	0,00	0,00
GNA	Manα1-3Man, Manα1-6Man	0,00	0,00	0,00	0,05
DBAI	High-man	51,96	39,33	74,44	92,17
CCA	Galactosylated N-glycans up to triantenna	0,00	0,00	0,12	0,79
Heltuba	Manα1-3Man	1,80	0,82	7,98	9,35
VVAII	Man, agalacto	0,00	0,00	0,00	0,00
rOrysata	Manα1-3Man, Highman, biantenna	0,02	0,00	1,29	1,96
rPALa	Man5, biantenna	0,00	0,00	12,18	13,23
rBanana	Manα1-2Manα1-3(6)Man	0,00	0,00	0,00	0,00
rCalsepa	Biantenna with bisecting GlcNAc	0,00	0,00	0,00	0,02
rRSL	αMan, α1-2Fuc (H), α1-3Fuc (Lex), α1-4Fuc (Lea)	0,00	0,00	0,00	0,00
rBC2LA	αMan, High-man	0,00	0,00	0,00	0,00
AOL	α1-2Fuc (H), α1-3Fuc (Lex), α1-4Fuc (Lea)	0,00	0,00	0,00	0,00
AAL	α1-2Fuc (H), α1-3Fuc (Lex), α1-4Fuc (Lea)	0,00	0,00	0,00	0,00
rAAL	α1-2Fuc (H), α1-3Fuc (Lex), α1-4Fuc (Lea)	44,81	38,10	61,45	62,14
rPAIIL	αMan, α1-2Fuc (H), α1-3Fuc (Lex), α1-4Fuc (Lea)	80,41	64,53	69,50	67,32

rPTL	α1-6Fuc	0,00	0,00	0,00	0,00
PSA	α1-6Fuc up to biantenna	3,04	0,99	5,19	10,64
LCA	α1-6Fuc up to biantenna	14,38	7,72	26,08	35,20
rAOL	α1-2Fuc (H), α1-3Fuc (Lex), α1-4Fuc (Lea)	0,00	0,00	36,50	36,73
rBC2LCN	Fuc α1-2Galβ1-3GlcNAc (GalNAc)	0,00	0,00	13,28	12,84
UEAI	α1-2Fuc	0,00	0,00	0,00	0,00
TJAII	α1-2Fuc	59,76	67,27	109,12	120,15
MCA	α1-2Fuc	0,00	0,00	0,31	1,45
FLAG-EW29Ch	Gal	0,15	0,63	11,70	13,71
PTLI	αGalNAc (A, Tn)	0,00	0,00	0,00	0,06
GSLIA4	αGalNAc (A, Tn)	0,00	0,00	0,00	0,00
rGC2	α1-2Fuc (H), αGalNAc (A), αGal (B)	0,00	0,00	0,00	0,00
GSLIB4	αGal (B)	0,00	0,00	4,69	4,58
rMOA	αGal (B)	0,00	0,00	0,00	0,00
EEL	αGal (B)	65,76	15,21	47,89	46,77
rPAIL	a,βGal, αGalNAc (Tn)	0,00	0,00	0,00	0,00
LEL	Polylactosamine, (GlcNAc)n	0,00	0,00	0,00	0,03
rCGL3	LacDiNAc	0,11	0,00	2,08	1,90
PNA	Galβ1-3GalNAc (T)	0,00	0,00	0,00	0,00
ACA	Galβ1-3GalNAc (T)	0,15	0,00	5,22	4,10
ABA	Galβ1-3GalNAc (T), GlcNAc	0,00	0,00	0,00	0,00
Jacalin	Galβ1-3GalNAc (T), GalNAcα (Tn)	0,04	0,00	0,16	0,54
HPA	αGalNAc (A, Tn)	23,98	5,12	15,35	14,19
VVA	a,βGalNAc (A, Tn, LacDiNAc)	4,97	1,26	15,10	16,75
DBA	a,βGalNAc (A, Tn, LacDiNAc)	0,00	0,00	0,00	0,00
SBA	a,βGalNAc (A, Tn, LacDiNAc)	0,19	0,18	11,64	16,48
rPPL	a,βGalNAc (A, Tn, LacDiNAc)	0,00	0,00	0,00	0,00
rCNL	a,βGalNAc (A, Tn, LacDiNAc)	0,11	0,00	0,16	0,00
rXCL	Core1,3, αGalacto N-glycan	0,00	0,00	0,00	0,00
VVA I	GalNAcβ1-3(4)Gal	91,18	79,52	91,81	87,66
WFA	Terminal GalNAc, LacDiNAc	0,00	0,00	0,00	0,00
rABA	Galβ1-3GalNAc (T), GlcNAc	0,00	0,00	0,00	0,00
rDiscoidin I	Gal	0,00	0,00	0,00	0,00
DBAIII	Maltose	88,91	106,58	145,11	135,76
rMalectin	Glcα1-2Glc	0,00	0,00	0,53	0,12

CSA	Rhamnose, Galα1-4Gal	0,00	0,00	0,00	0,00

Fluorescence in Situ Hybridization (FISH)

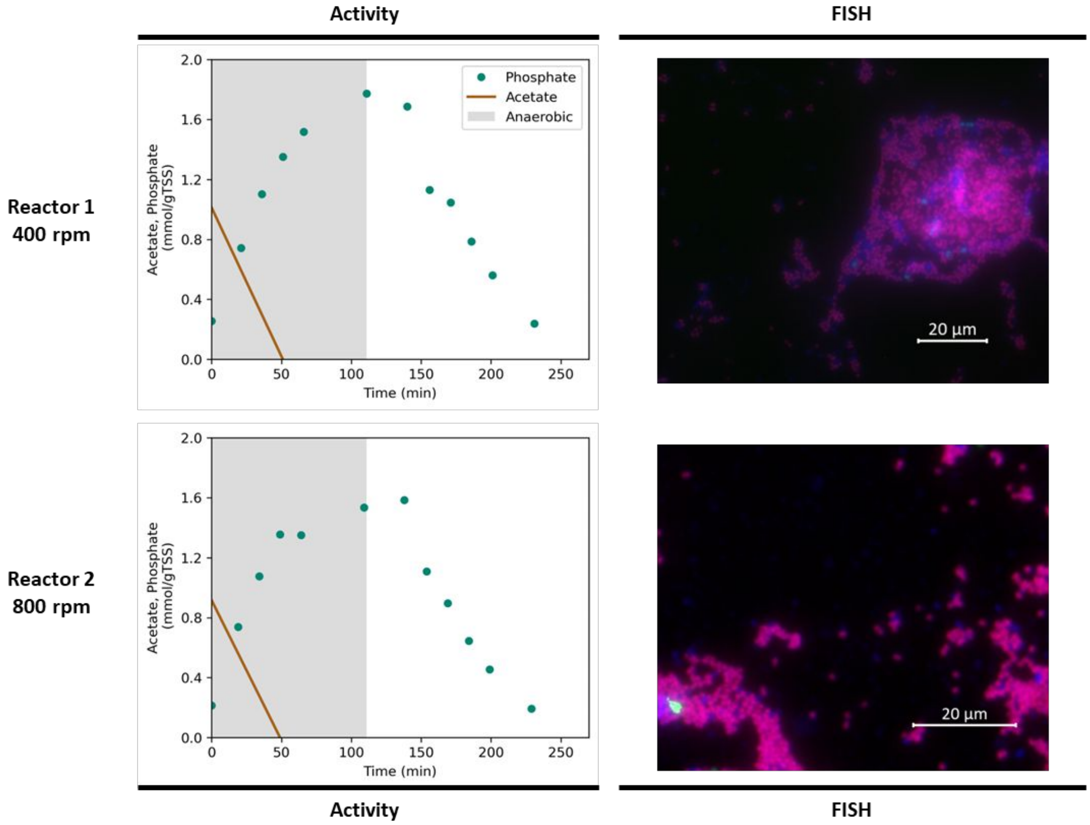

Figure S1. *Reactor characteristics for the enrichments with the impeller rotating at 400 (top) and 800 (bottom) rpm at steady state. Each panel presents the activity test of a cycle by showing the concentrations of phosphate and acetate (mmol/gTSS) (left) and the FISH image of the PAO enrichment, with PAOmix probes (targeting "Ca. Accumulibacter," in red), GAOmix probes (targeting "Ca. Competibacter" in green), and EUBmix probes (targeting all bacteria, in blue). Magenta color represents the overlap of "Ca. Accumulibacter" (red) and eubacteria (blue); cyan color represents the overlap of "Ca. Competibacter" (green) and eubacteria (blue).*

GS-MS spectrum from glycosyl analysis

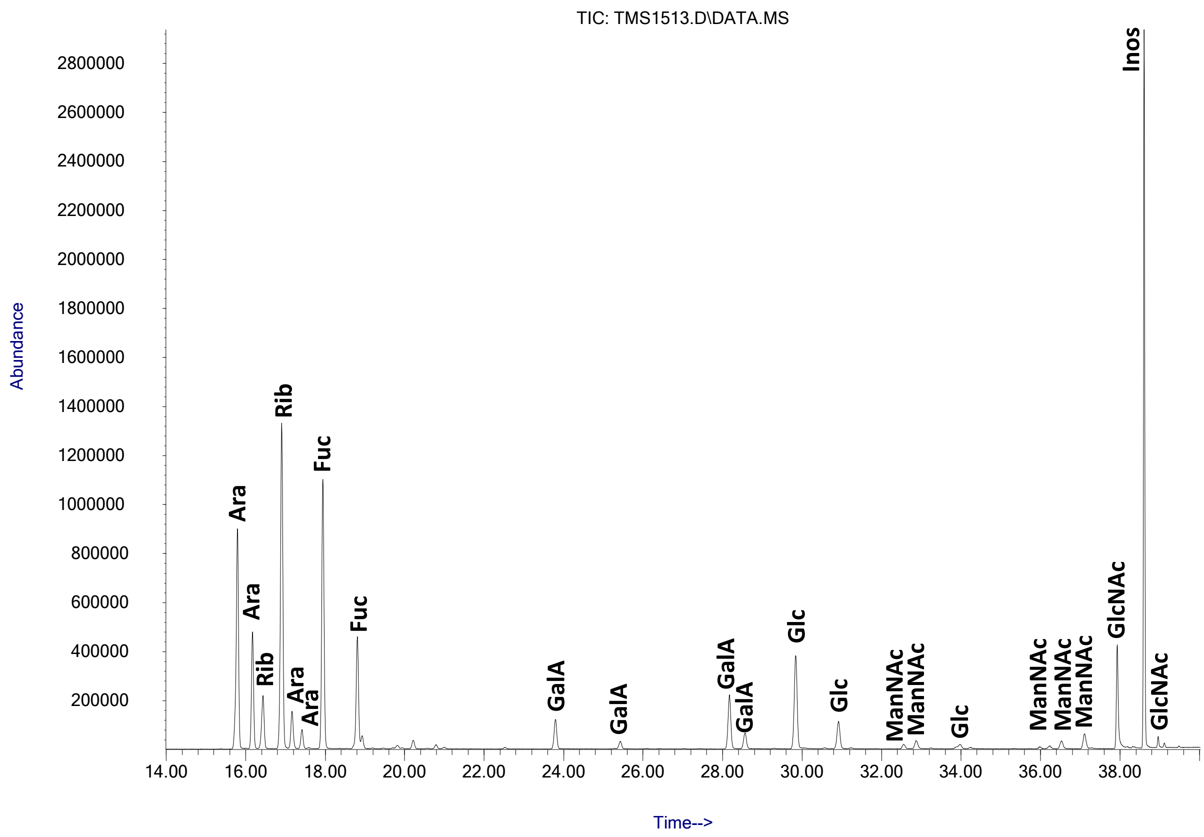

Figure S2*: Chromatogram of Standard 1 used for composition analysis of TMS methyl glycosides.*

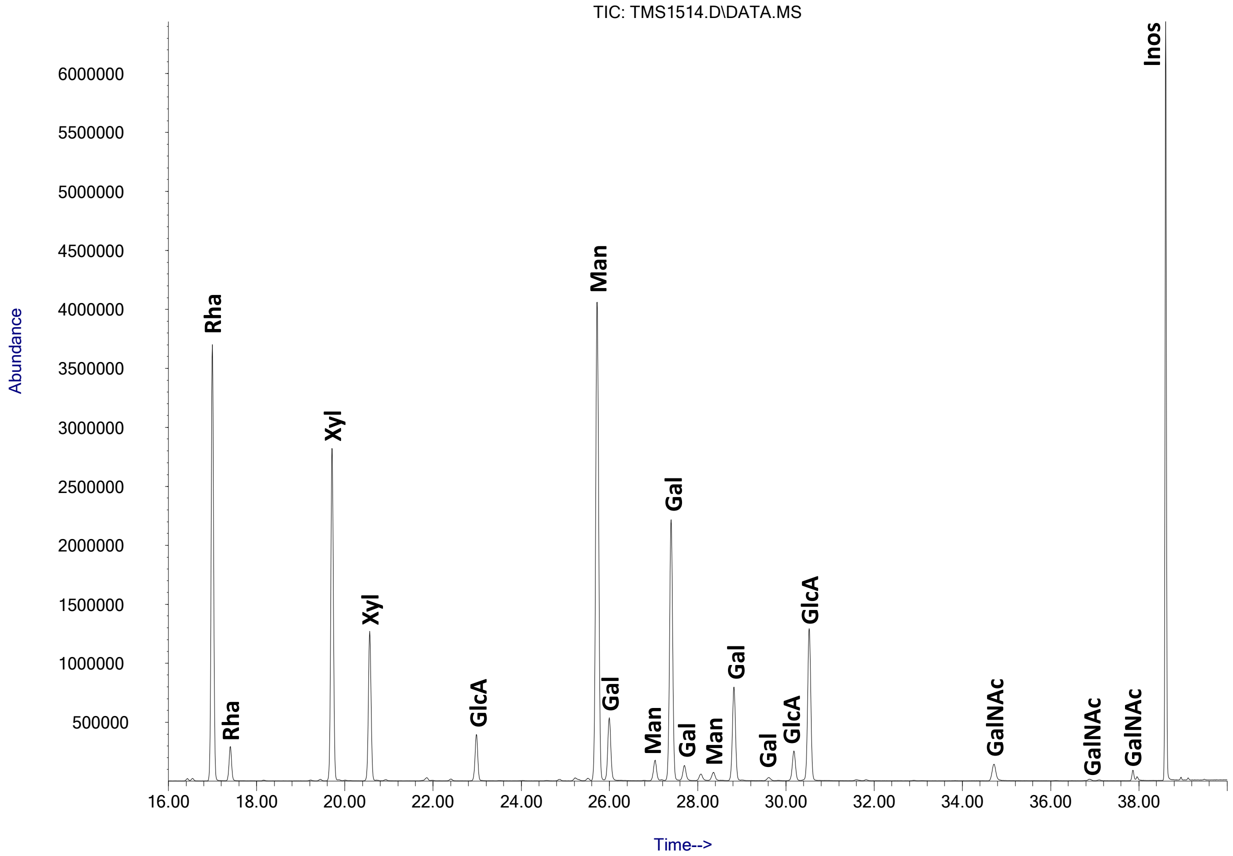

***Figure S3**: Chromatogram of Standard 2 used for composition analysis of TMS methyl glycosides.*

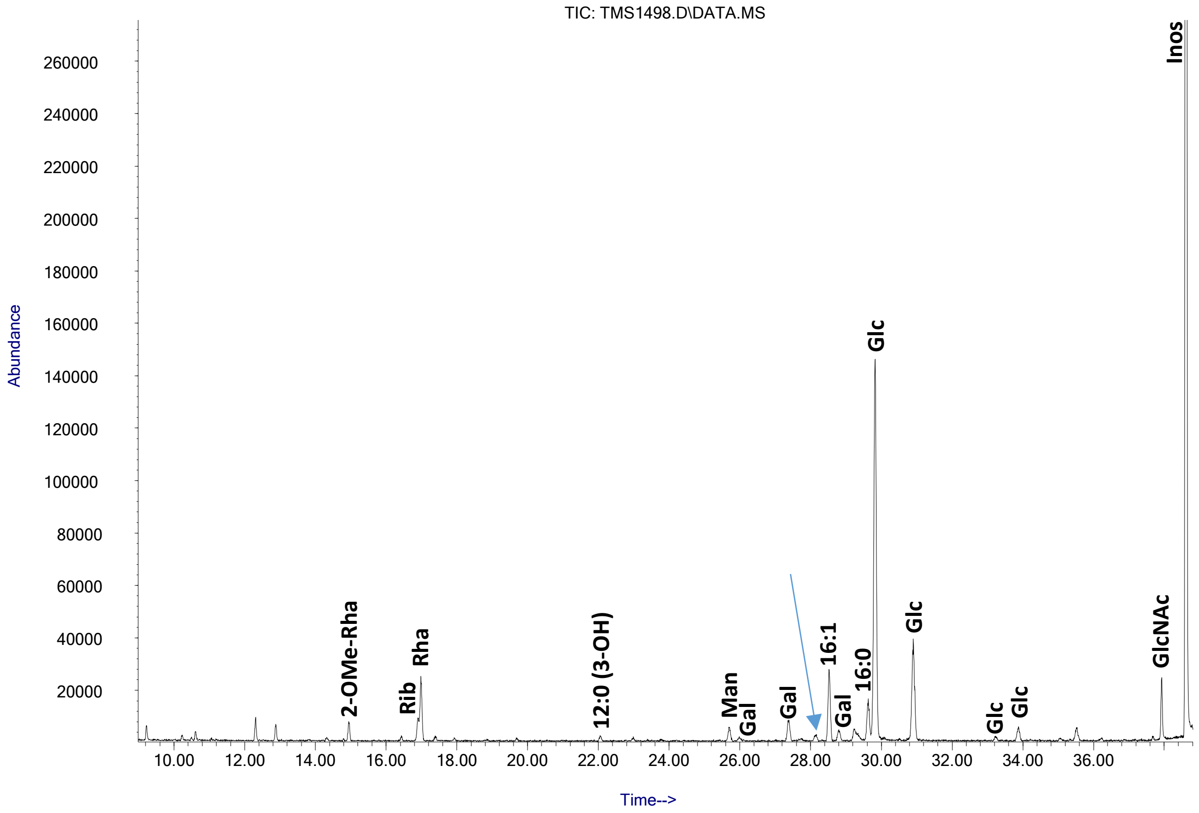

***Figure S4**: Chromatogram of EPS sample sample 1used for composition analysis of TMS methyl glycosides (the blue arrow points to QuiNAc).*

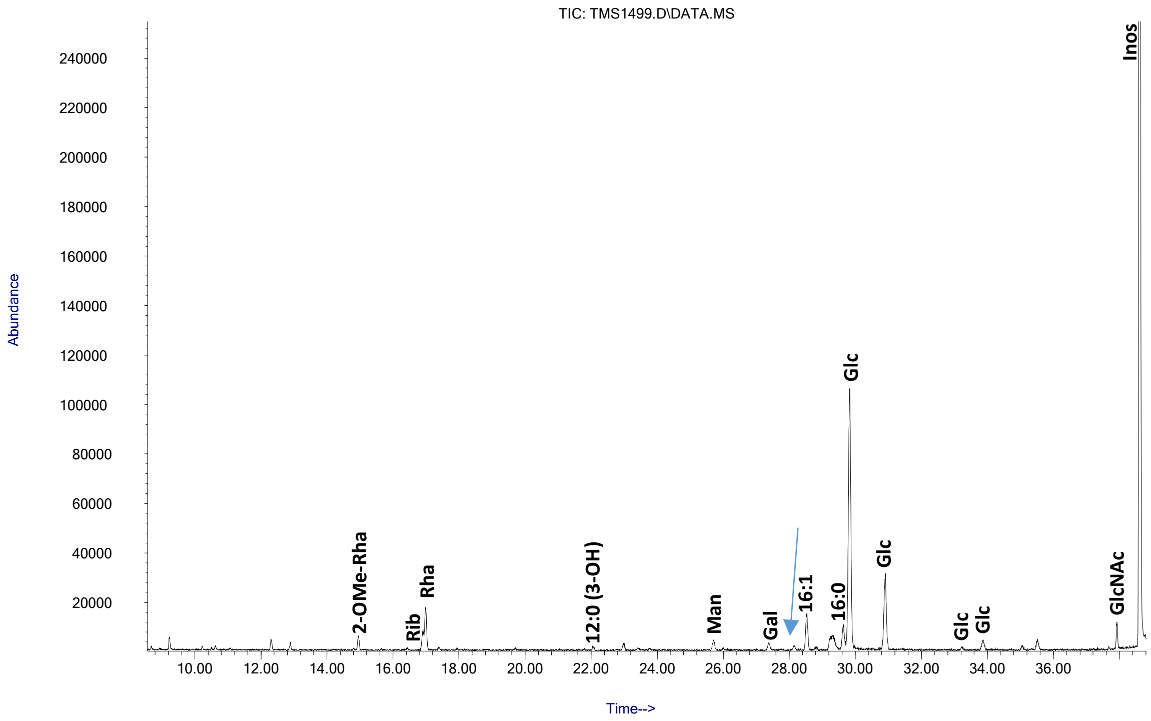

Figure S5: *Chromatogram of EPS sample sample 2 used for composition analysis of TMS methyl glycosides (the blue arrow points to QuiNAc).*
